# Genetic variation of RNF145 gene and blood lipid levels in Xinjiang population, China

**DOI:** 10.1038/s41598-021-85503-z

**Published:** 2021-03-16

**Authors:** Jing Ming, Xian Wei, Min Han, Dilare Adi, Jialin Abuzhalihan, Yong-Tao Wang, Yi-Ning Yang, Xiao-Mei Li, Xiang Xie, Zhen-Yan Fu, Min-Tao Gai, Yi-Tong Ma

**Affiliations:** 1 Department of Cardiology, First Affiliated Hospital of Xinjiang Medical University, Urumqi, 830054 People’s Republic of China; 2 Xinjiang Key Laboratory of Cardiovascular Disease Research, Urumqi, 830054 People’s Republic of China

**Keywords:** Cardiology, Cardiovascular biology, Genetics research

## Abstract

Dyslipidemia is one of the main risk factors for coronary heart disease (CHD). The E3 ubiquitin ligase which is encoded by the ring finger protein 145 (RNF145) gene is very important in the mediation of cholesterol synthesis and effectively treats hypercholesterolemia. Thus, the purpose of the present research is to investigate the connection between the polymorphism of the RNF145 gene and cholesterol levels in the populations in Xinjiang, China. A total of 1396 participants (Male: 628, Female: 768) were included in this study for genetic analysis of RNF145 gene, and we used the modified multiple connection detection response (iMLDR) technology to label two SNPs (rs17056583, rs12188266) of RNF145 genotyping. The relationship between the genotypes and the lipid profiles was analyzed with general linear model analysis after adjusting confounding variables. Through the analysis of the two SNPs in RNF145 gene, we discovered that both rs17056583 and rs12188266 were related to total cholesterol (TC) and low-density lipoprotein cholesterol (LDL-C) concentrations (All P < 0.001). In addition, the association of rs17056583 and rs12188266 with lipid profiles concentrations is still statistically significant after multivariate adjustment of sex, age, smoking, obesity, drinking, diabetes, hypertension and lipid profiles. Meanwhile, we also found that rs17056583 was associated with high triglycerides concentrations before and after adjustment (All P < 0.001). Our study shows that both rs17056583 and rs12188266 SNPs of RNP145 gene are related to TC and LDL-C concentrations in Xinjiang population.

## Introduction

In recent years, coronary heart disease (CHD) is one of the diseases with the highest morbidity and mortality in both developed and developing countries^1,2^. Different degrees of coronary stenosis and myocardial ischemia due to lipid metabolism disorders are the most important pathological basis of CHD. Dyslipidemia, which is characterized by elevated low-density lipoprotein cholesterol (LDL-C) or triglycerides (TG), is a crucial risk factor for CHD. Long-term deposition of LDL-C in blood vessels causes CHD, and leads to myocardial infarction, eventually^3,4^. Blood lipid metabolism is a complex network regulation system. Plasma cholesterol levels are affected by genetic variation and environmental factors. Cholesterol synthesis and metabolism are regulated by multiple genes^5–7^. Also, the genetic constitution of each individual has a very large effect on plasma cholesterol levels, such as single nucleotide polymorphisms (SNPs)^8–10^. At present, the effects of many genetic variations on cholesterol levels are unclear, and the lipid-lowering drugs can not completely adjust plasma blood lipid levels to expectation. Hence, we try to explore more new possible strategies for lipid-lowering treatment.

In cholesterol biosynthesis, polytopic membrane glycoprotein 3-hydroxy-3-methylglutaryl coenzyme A reductase (HMGCR), where endoplasmic reticulum (ER) resides, is the rate-limiting enzyme, catalyzing the formation of mevalonate^11^. Statins, as competitive inhibitors of HMGCR, reduce plasma cholesterol levels and delay the development of cardiovascular disease on the basis of this physiological process^12^. However, statin resistance exists during clinical usage, which would attenuate their benefits. Therefore, it is of great biological significance and clinical value to study new genes on regulating HMGCR in cardiovascular diseases.

The ring finger protein 145 (RNF145) gene encodes an ER-resident E3 ubiquitin ligase. Cook et al.^13,14^ found that the RNF145 gene transcription is regulated by the sterol-responsive liver X receptor (LXR) family of transcription factors, which transcriptionally activates cholesterol efflux pumps (ABCA1, ABCG1) and the IDOL E3 ubiquitin ligase^15–17^. The RNF145 is the main ubiquitin ligase that promotes the degradation of HMGCR. Studies have shown that RNF145 can inhibit endogenous cholesterol synthesis and effectively treat hypercholesterolemia. It is not only an important negative factor in the regulation of cholesterol synthesis, but also a new target for the regulation of blood lipids^18^. Therefore, this study is to explore the differences in distribution characteristics of RNF145 gene polymorphisms in Xinjiang population in China, and the relationships between the gene polymorphisms and blood lipid levels.

## Methods

### Subjects

From January 2016 to December 2019, 1440 participants were selected from the patients admitted to the Heart Center of the First Affiliated Hospital of Xinjiang Medical University for research.

Finally, the present study included 1396 subjects (male: n = 628; female: n = 768) who had complete data on RNF145 genotype, and whole subjects are the residents in Xinjiang Uygur Autonomous Region of China. Exclusion criteria included those suffering from impaired malignancy, connective tissue disease, concomitant valvar heart disease, renal function, valvular disease or chronic inflammatory disease, pancreatic disease, fatty liver, cirrhosis, hepatitis. Moreover, subjects should also be free from thyroid disease, or any history of taking lipid-lowering drugs. Hypertension was defined as a systolic blood pressure ≥ 140 mmHg and/or a diastolic blood pressure ≥ 90 mmHg at least on two distinct occasions^27^. Diabetes mellitus was defined as two fasting plasma glucose (FPG) level ≥ 7.0 mmol/L^28^. The following information was collected: age, gender, hypertension, diabetes, total cholesterol (TC), TG, high-density lipoprotein cholesterol (HDL-C), and LDL-C. According to the 2016 Chinese Guidelines for the Management of Dyslipidemia in Adults^29^. high TC was defined as TC ≥ 6.22 mmol/L, high LDL-C was defined as LDL-C ≥ 4.14 mmol/L, low HDL-C was defined as HDL-C ≤ 1.04 mmol/L, and high TG was defined as TG ≥ 2.26 mmol/L.

### Collection of clinical data and detection of blood biochemical indicators

Subjects’ height and weight were measured. Medical history and living habits including smoking, drinking, diabetes, hypertension, were obtained in detail. The blood biochemical indexes including blood urea nitrogen (BUN), creatinine, uric acid (UA), glucose, TC, TG, LDL-C, and HDL-C, were measured and reported by the Medical Laboratory Center.

### Ethical approval of research protocols

The study was approved officially by the Ethics Committee of the first affiliated Hospital of Xinjiang Medical University (Urumqi, China) and carried out with standards of Helsinki Declaration. Written informed consent and permission to collect relevant clinical data were obtained from the participants.

### SNP selection and genotyping

The human RNF145 gene is on the 33.3 position of the long arm of chromosome 5, and contains 16 exons and 15 introns. 5 ml of venous blood were extracted from the subjects, and were added anticoagulant, and 3000 rpm or 3000 revolution per minute centrifugation was used to separate plasma and red blood cells which were saved in a place with − 80 °C or − 80 degrees of Celsius, and we used whole blood genome extraction kit, according to the instructions for DNA extraction. In this study, we screened the data for the Tag SNPs on the International HapMap Project website (http://www.hapmap.org/). Using the Haploview 4.2 software and International HapMap Project website phase I&II database, we obtained two tag SNPs (rs17056583, rs12188266) for Chinese Han using minor allele frequency (MAF) ≥ 0.05 and linkage disequilibrium patterns with r^2^ ≥ 0.8 as a cutoff. With unknowing the clinical data of the patients, SNP genotyping was carried out by using the improved multiplex linkage detection reaction (iMLDR) technique, and 10% of the genotyping samples were repeated to monitor the quality of genotyping.

### Statistical methods

We used SPSS23.0 software to analyze data. The data were expressed as mean ± standard deviation. Comparison between the two groups were performed with the t-test, Chi-squared test and two-way ANOVA, respectively. The Hardy–Weinberg test was used to test whether the subjects are typical or not. The relationship between the single nucleotide polymorphism model and the lipid profile was analyzed with general linear model analysis after adjusting confounding variables. P < 0.05 is statistically significant.

### Ethics approval and consent to participate

The study was approved by the Ethical Review Board of The First Affiliated Hospital of Xinjiang Medical University. Written informed consent was obtained from all enrolled patients.

## Result

### Clinical and metabolic characteristics of study subjects

A total of 1396 participants were included in this study, and divided into two groups (male: 628; female: 768). Table 1 represents the clinical and metabolic features of the male and female subjects, respectively. According to the analysis, compared with females, body mass index (BMI), the plasma concentrations of UA, creatinine, TG, TC, and LDL-C, and the frequency of smoking and drinking were higher in males (All P < 0.05). Furthermore, on BUN and HDL-C, we found no significant differences between men and women (All P > 0.05).

**Table 1:** Clinical and metabolic characteristics of subjects.

Factors	Total (n = 1396)	Male (n = 628)	Female (n = 768)	t/χ2	*P* value
Age (years)	34.12 ± 5.20	33.69 ± 5.55	34.46 ± 4.89	− 1.376	0.170
BMI (kg/m^2^)	24.61 ± 4.10	25.43 ± 4.01	23.93 ± 4.05	3.437	0.001
UA (μmol/L)	245.87 ± 98.45	300.30 ± 99.14	201.36 ± 64.38	10.782	< 0.001
BUN (mmol/L)	4.46 ± 3.17	4.65 ± 1.36	4.30 ± 4.10	1.020	0.308
SBP (mmHg)	121.96 ± 16.52	125.59 ± 14.93	118.99 ± 17.19	3.780	< 0.001
DBP (mmHg)	74.84 ± 13.56	77.41 ± 13.37	72.73 ± 13.99	3.251	0.001
Cr (μmol/L)	66.47 ± 24.30	78.00 ± 25.45	57.04 ± 18.67	8.603	< 0.001
TG (mmol/L)	1.39 ± 1.63	1.88 ± 2.24	0.98 ± 0.61	4.828	< 0.001
TC (mmol/L)	3.86 ± 1.60	4.13 ± 1.72	3.65 ± 1.47	2.792	0.005
HDL-C (mmol/L)	1.05 ± 0.40	1.02 ± 0.40	1.06 ± 0.40	− 0.924	0.356
LDL-C (mmol/L)	2.36 ± 1.45	2.60 ± 1.53	2.17 ± 1.35	2.763	0.005
Diabetes	68 (4.9)	44 (7.0)	24 (3.1)	3.906	0.048
Hypertension	208 (14.9)	120 (19.1)	88 (11.5)	7.658	0.103
Smoking (%)	376 (26.9)	340 (54.1)	36 (4.6)	107.334	< 0.001
Drinking (%)	168 (12.0)	160 (25.4)	8 (1.04)	48.719	< 0.001

Continuous variables are expressed as mean ± SD. Categorical variables are expressed as percentages.

*BMI* body mass index, *TG* triglyceride, *TC* total cholesterol, *HDL-c* high density lipoprotein, *LDL-c* low density lipoprotein, *DBP* diastolic blood pressure, *SBP* systolic blood pressure, *UA* uric acid.

### RNF145 genotype and allele frequencies

1. Hardy–Weinberg equilibrium test: the χ^2^ values of genotype distribution in the sample were 0.953 and 0.635, respectively. The results of Hardy–Weinberg equilibrium test show that the population can be regarded as a typical sample.
2. The distribution of genotypes and alleles of SNPs for RNF145 gene were shown in Table 2. For rs17056583 genotypes, we discovered that there was significant difference between males and females in the dominant model (GG vs CC + GC), recessive model (CC vs GG + GC), and allele frequency (P = 0.016, P = 0.004 and P = 0.002). Compared with males, the G allele of rs17056583 in female participants were significantly higher (men: 908; women: 1260). For the rs12188266 genotypes, the distribution of the recessive model (GG vs AA + AG) and allele frequency are different from male and female participants (P < 0.001, P = 0.007). We observed that there were no significant differences between males and females in additive model (AG vs GG + AA) and dominant model (AA vs GG + AG) (All P > 0.05). The A allele of rs12188266 was higher in female participants than in male participants (men: 928; women: 1294).

**Table 2:** Distribution of SNPs of RNF145 gene for study population.

Genotype	Model	Total (n, %)	Male (n, %)	Female (n, %)	P value	P value
rs17056583	**Genotype**					
	CC	72 (5.2)	56 (8.9)	16 (2.1)	10.952	0.004
	GC	480 (34.4)	236 (37.6)	244 (31.8)		
	GG	844 (60.5)	336 (53.5)	508 (66.1)		
	**Rec**					
	CC	72 (5.2)	56 (8.9)	16 (2.1)	8.246	0.004
	GC + CC	1324 (94.8)	572 (91.1)	752 (97.9)		
	**Dom**					
	GG	844 (60.5)	336 (53.5)	508 (66.1)	5.775	0.016
	GC + CC	552 (39.5)	292 (46.5)	260 (33.9)		
	**Add**					
	GC	480 (34.4)	236 (37.6)	244 (31.8)	1,292	0.256
	CC + GG	916 (65.6)	392 (62.4)	524 (68.2)		
	**Allele**					
	G	2168 (77.7)	908 (72.3)	1260 (82.0)	9.440	0.002
	C	624 (22.3)	348 (27.7)	276 (18.0)		
rs12188266	**Genotype**					
	GG	76 (5.4)	64 (10.2)	12 (1.6)	12.744	0.002
	AG	448 (32.1)	200 (31.8)	248 (32.3)		
	AA	872 (62.5)	364 (58.0)	508 (66.1)		
	**Rec**					
	GG	76 (5.4)	64 (10.2)	12 (1.6)	12.492	< 0.001
	AG + AA	1320 (94.6)	564 (89.8)	756 (98.4)		
	**Dom**					
	AA	872 (62.5)	364 (58.0)	508 (66.1)	2,467	0.116
	AG + GG	524 (37.5)	264 (42.0)	260 (33.9)		
	**Add**					
	AG	448 (32.1)	200 (31.8)	248 (32.3)	0.008	0.929
	GG + AA	948 (67.9)	428 (68.2)	520 (67.7)		
	**Allele**					
	A	2192 (78.5)	928 (73.9)	1264 (82.3)	7.235	0.007
	G	600 (21.5)	328 (26.1)	272 (17.7)		

### The relationship between RNF145 genotypes and lipid profiles

**Figure 1:**
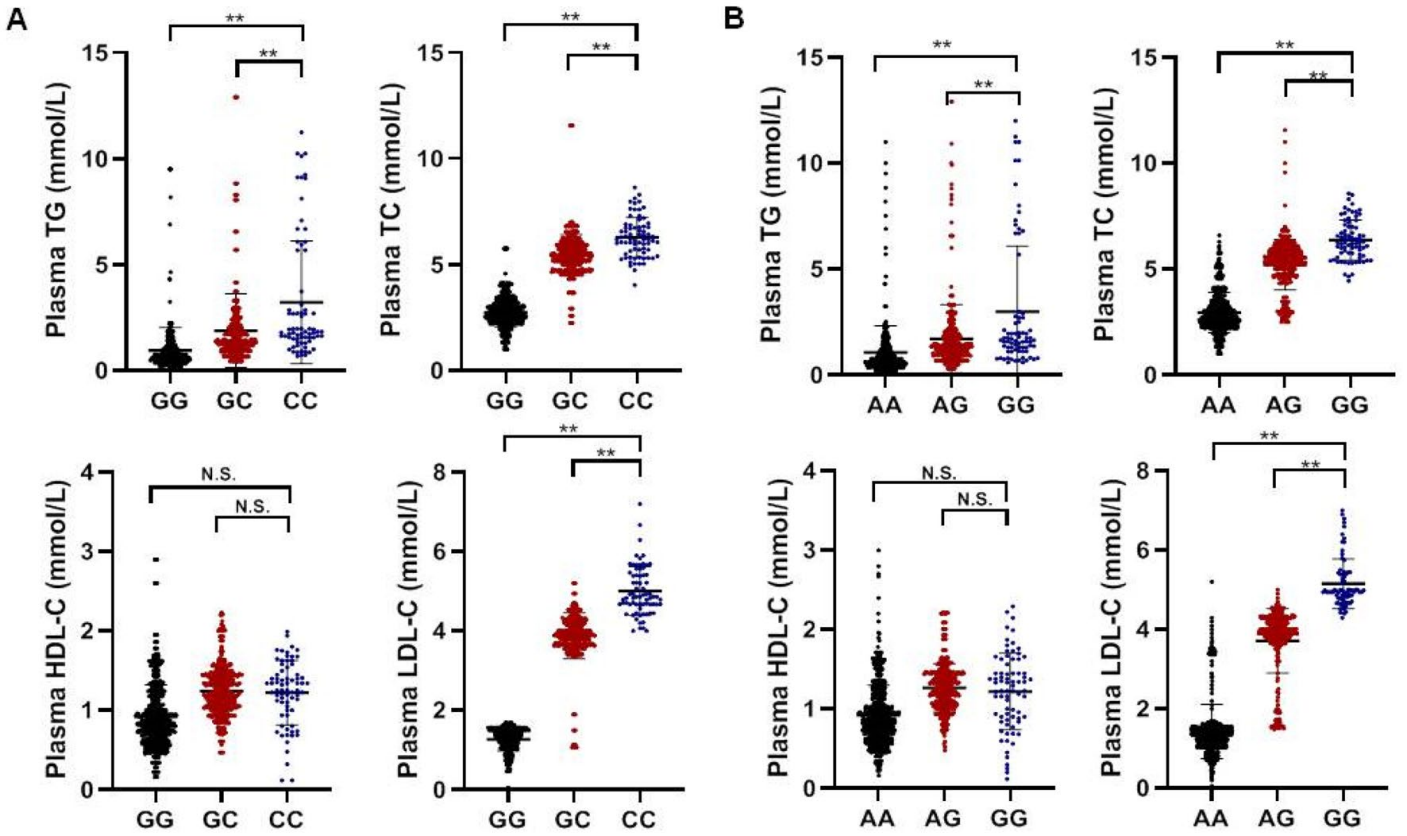
(**A**) Influence of the RNF145 gene rs17056583 on the lipid profile. n = 1396. Values are means ± SD. (**B**) Influence of the RNF145 gene rs12188266 on the lipid profile. n = 1396. Values are means ± SD. *P < 0.05, **P < 0.001, N.S. indicates no significance.

1. The plasma TC concentrations and LDL-C concentrations was significantly higher in participants with CC genotype than that in participants with GC or GG genotype (both P < 0.001, Fig. 1A). Using general linear model analysis, we discovered the rs17056583 was associated with TC, TG, and LDL-C concentrations in a dominant model, additive model, and recessive model (All P < 0.05). With the multivariate adjustments to the main covariates (sex, age, smoking, obesity, drinking, diabetes, hypertension and lipid profiles), the correlation remains significant (P < 0.05; Table 3).
2. The plasma TC concentrations and LDL-C concentrations were greater in participants with GG genotype compared with participants with AG or AA genotypes (both P < 0.001, Fig. 1B). By analyzing the three models, we observed that the rs12188266 was significantly associated with plasma TC and LDL-C concentrations (All P < 0.05). After multivariate adjustment to the key co-variants (sex, age, smoking, obesity, drinking, diabetes, hypertension and lipid profiles), the correlation remains significant in additive model and recessive model (All P < 0.05). Furthermore, we found that rs12188266 was significantly associated with HDL-C levels (P < 0.05) and the difference remained statistically significant in a dominant and an additive model after multivariate adjustment (P < 0.05; Table 4).

**Table 3:** Associations between rs17056583 and lipid parameters.

rs17056583	Homozygous for wild allele (G)	Heterozygous (GC)	Homozygous for rare allele (C)	Model 1			Model 2		
				P (Dom)	P (Add)	P (Rec)	P (Dom)	P (Add)	P (Rec)
TG	0.96 ± 1.10	1.88 ± 1.75	3.11 ± 3.31	< 0.001	< 0.001	0.018	0.033	0.034	0.037
TC	2.76 ± 0.70	5.44 ± 0.98	6.29 ± 1.04	< 0.001	0.002	< 0.001	< 0.001	< 0.001	< 0.001
HDL-C	0.92 ± 0.40	1.24 ± 0.32	1.18 ± 0.41	< 0.001	< 0.001	0.073	0.873	0.459	0.235
LDL-C	1.27 ± 0.29	3.87 ± 0.62	5.10 ± 0.60	< 0.001	< 0.001	< 0.001	< 0.001	< 0.001	< 0.001

*TC* total cholesterol; *LDL-C* low density lipoprotein-cholesterol; *HDL-C* high density lipoprotein-cholesterol; *TG* triglycerides; *Dom* dominant model; *Rec* recessive model; *Add* additive model; *Model 1* unadjusted model; *Model 2* analysis of covariance adjusted for sex, age, smoking, obesity, drinking, diabetes, hypertension and lipid profiles.

**Table 4:** Associations between rs12188266 and lipid parameters.

rs12188266	Homozygous for wild allele (A)	Heterozygous (AG)	Homozygous for rare allele (G)	Model 1			Model 2		
				P (Dom)	P (Add)	P (Rec)	P (Dom)	P (Add)	P (Rec)
TG	1.07 ± 1.26	1.71 ± 1.65	3.06 ± 3.22	< 0.001	0.033	0.003	0.814	0.610	0.208
TC	2.95 ± 0.99	5.21 ± 1.23	6.39 ± 0.98	< 0.001	0.02	< 0.001	0.443	0.005	< 0.001
HDL-C	0.92 ± 0.39	1.26 ± 0.31	1.18 ± 0.46	< 0.001	< 0.001	0.003	0.016	< 0.001	0.292
LDL-C	1.44 ± 0.70	3.68 ± 0.92	5.15 ± 0.55	< 0.001	< 0.001	< 0.001	0.775	< 0.001	< 0.001

*TC* total cholesterol; *LDL-C* low density lipoprotein-cholesterol; *HDL-C* high density lipoprotein-cholesterol; *TG* triglycerides; *Dom* dominant model; *Rec* recessive model; *Add* additive model; *Model 1* unadjusted model; *Model 2* analysis of covariance adjusted for sex, age, smoking, obesity, drinking, diabetes, hypertension and lipid profiles.

## Discussion

In this study, we genotyped two SNPs in the RNF145 gene, and found that the mutation in the RNF145 gene was linked to cholesterol levels of the population in Xinjiang. Our research suggests that the RNF145 gene may be a target to lower cholesterol levels and treat hypercholesterolemia.

Xinjiang Province locates in the northwest of China. Compared with inner areas of China, there are some differences in ecological environment, economic conditions, genetic backgrounds, lifestyles and eating habits in Xinjiang. As to Xinjiang populations, the prevalence of dyslipidemia of them is higher than the people who live in other parts of China, and is different from one another in each ethnic group^30,31^. The main reason of these differences may be, firstly, the different genetic backgrounds. Actually, people live in Xinjiang mostly eat pasta, dairy products, milk tea (mainly containing sodium), beef and mutton as their staple food, and consume less fruits and vegetables. Therefore, it is of great significance to study on blood lipid levels in this population in Xinjiang.

Before this study, there were few researches related to the connection between genetic variation of RNF145 gene and lipid distribution in China. A genome-wide association study (GWAS) conducted by Gieger et al.^32^ identified a SNP in the RNF145 gene which was significantly associated with mean platelet volume (MPV). In addition, Bowes et al.^33^ revealed in their study that a novel polymorphism in the RNF145 gene is significantly associated with the genetics of psoriatic arthritis, but the full pathogenetic mechanism is not yet elucidated. Furthermore, several lines of evidence suggest that plasma lipid levels are influenced by genetic factors. Recently, a GWAS has also showed that the RNF145 gene polymorphism is significantly associated with plasma lipid levels. Richardson et al.^34^ reported that the rs55801554 in human RNF145 gene was associated at P < 5 × 10^−8^ with HDL-C (4 × 10^–10^) and apolipoprotein A (2 × 10^–9^), which means the RNF145 meet genome-wide significance threshold. We obtained the information for studied SNPs in HaploReg, and found that both rs17056583 and rs12188266 showed overlap in promoter or enhancer regions. Among the two SNPs, the rs12188266 regulates the expression of RNF145 gene in peripheral blood^35^. Our research demonstrates that rs17056583, rs12188266, and plasma TC, LDL-C concentrations are correlated according to the analysis of dominant, additive, and recessive models. The significant association with plasma TC and LDL-C concentrations in the additive and recessive models of rs17056583, rs12188266 were retained after adjustment to covariates such as gender, age, smoking, obesity, drinking, diabetes, hypertension, and lipid profile. Contrasted with the G allele, the plasma TC and LDL-C concentrations of individuals carrying the C allele of rs17056583 were significantly higher. Lipid metabolism is a complex regulatory network system, and lipid abnormalities characterized by LDL-C in the lipid profile are also important risk factors for hypercholesterolemia and CAD. Therefore, the CC genotype of rs17056583 may be a dangerous genetic marker that causes elevated plasma cholesterol levels. People who carry the C allele are more likely to develop dyslipidemia than those who carry the G allele of the rs17056583. We also observed that the A allele of rs12188266 had much lower plasma TC levels and LDL-C levels compared to the G allele. Therefore, the result indicates that people who carry the A allele of rs12188266 have a reduced risk of dyslipidemia compared with the people who carry the G allele. Besides, we also observed significant differences between TG levels and the rs17056583 of the RNF145 gene, which indicates that there may be also a correlation between the RNF145 gene and TG concentrations. However, the connection between TG levels and the RNF145 gene needs further research. These findings suggest that RNF145 gene polymorphisms may predict lipid profile in Xinjiang population.

The mechanism which RNF145 participates in cholesterol biosynthesis is not fully understood. RNF145 is essentially an unstable E3 ubiquitin ligase. The endogenous RNF145 is regulated by the state of sterols. When sterols are depleted, the expression of RNF145 mRNA and HMGCR mRNA will increase^26^. RNF145 was collected into insulin-induced genes (Insigs) when sterol was sufficient, which promoted ubiquitination regulation of HMGCR and accelerated degradation of sterol, which can reduce cholesterol levels^18^. Meanwhile, RNF145 also causes the ubiquitination of SCAP and interferes with the combination of SCAP and COPII, which can inhibit the maturity of SREBP-2 and reduce the cholesterol level^14^.

In the process of cholesterol synthesis, the essential function of the RNF145 was ubiquitination. Based on the previous researches on ubiquitination, we found that the ubiquitin–proteasome system is involved in all cellular processes^19^. The function of this system mostly depends largely on E3 ubiquitin ligase^20^. However, we found that only a few E3s are directly related to cholesterol metabolism. According to previous studies, gp78 (also known as autocrine motility factor receptor [AMFR]), TRC8 (translocation in renal carcinoma chromosome 8) and MARCH6 (membrane-associated RING-CH-type finger 6) are E3 ubiquitin ligases located in ER, which promotes the HMGCR degradation in hepatocytes, and gp78 plays a major role among them^21–23^. Tsai et al.^24^ found that in primary mouse embryonic fibroblasts (MEFs) deficient in gp78, the sterol-induced degradation of HMGCR persists. Interestingly, the study by Jiang et al.^18^ found that knocking out the RNF145 gene alone had little influence on the degradation of HMGCR in Chinese hamster ovary (CHO) cells. However, RNF145 and gp78 genes were knocked out in CHO cells at the same time, the sterol-induced degradation of HMGCR was dramatically blunted.

Therefore, the RNF145, like gp78, is the main ubiquitin ligase that promotes the degradation of HMGCR, and they are functionally redundant. Erdenbat et al.^25^ first demonstrated the correlation between gp78 gene SNP rs2440472 and CAD in 2014. Thus, we speculate that there may be a correlation between the RNF145 gene and dyslipidemia. Among all the E3 ubiquitin ligases that we have found, RNF145 is the only protein used to inhibit the expression of genes, contained in cholesterol biosynthesis and reduce serum cholesterol levels. Therefore, RNF145 is an important protein that regulates lipid metabolism.

RNF145 is an important negative regulator of cholesterol biosynthesis. However, at present, there are few studies on the relationship between RNF145 and cardiovascular diseases. Our study firstly reports that RNF145 gene polymorphisms are associated with lipid profiles, which indicate that RNF145 may play a crucial role in the process of CHD. However, further studies are required to suggest RNF145 as a novel target of CHD treatment and prevention.

## Conclusions

According to the above results, in the study of the relationship between RNF145 gene polymorphisms and blood lipid levels, there are statistically significant differences in blood lipid levels among different genotypes in the population of Xinjiang.

## Data Availability

The data will not be shared, since part of the data is being reused by another study.

## Abbreviations

CHD: Coronary heart disease
HMGCR: 3-Hydroxy-3-methylglutaryl coenzyme A reductase
RNF145: Ring finger protein 145
HDL: High-density lipoprotein
LDL: Low-density lipoprotein
TC: Total cholesterol
TG: Triglyceride

## References

[1] Kreisberg RA, Oberman A (2002). Clinical review 141: Lipids and atherosclerosis: Lessons learned from randomized controlled trials of lipid lowering and other relevant studies. J. Clin. Endocrinol. Metab..

[2] Roger VL, Go AS, Lloyd-Jones DM (2011). Heart disease and stroke statistics-2011 update: A report from the American Heart Association. Circulation.

[3] Silbemagel G, Fader G, Renner W (2009). The relationships of cholesterol metabolism and plzsma plant sterols with the severity of coronary artery disease. J. Lipid. Res..

[4] Pilia G, Chen WM, Scuteri A (2006). Heritability of cardiovascular and personality traits in 6148 Sardinians. PLoS Genet..

[5] Heller DA, de Faire U, Pedersen NL (1993). Genetic and environmental influences on serum lipid levels in twins. N. Engl. J. Med..

[6] Sv DMC, Kanaan S, Chung KH (2011). Environmental factors, familial aggregation and heritability of total cholesterol, low density lipoprotein-cholesterol and high density lipoprotein-cholesterol in a Brazilian population assisted by the Family Doctor Program. Public Health.

[7] Mackay J, Mensah GA, Mackay J (2004). The atlas of heart disease and stroke. Un Chronicle..

[8] Aulchenko YS, Ripatti S, Lindqvist I, Boomsma D, Heid IM, Pramstaller PP (2009). Loci influencing lipid levels and coronary heart disease risk in 16 European population cohorts. Nat. Genet..

[9] Edmondson AC, Braund PS, Stylianou IM, Khera AV, Nelson CP, Wolfe ML (2011). Dense genotyping of candidate gene loci identifies variants associated with high-density lipoprotein cholesterol. Circ. Cardiovasc. Genet..

[10] Kathiresan S, Willer CJ, Peloso GM, Demissie S, Musunuru K, Schadt EE (2009). Common variants at 30 loci contribute to polygenic dyslipidemia. Nat. Genet..

[11] Goldstein JL, Brown MS (1990). Regulation of the mevalonate pathway. Nature.

[12] Kuulasmaa K, Tunstallpedoe H, Dobson A (2000). Estimation of contribution of changes in classic risk factors to trends in coronary-event rates across the WHO MONICA Project populations. Lancet.

[13] Cook EC, Nelson JK, Sorrentino V (2017). Identification of the ER-resident E3 ubiquitin ligase RNF145 as a novel LXR-regulated gene. PLoS ONE.

[14] Zhang L, Rajbhandari P, Priest C (2017). Inhibition of cholesterol biosynthesis through RNF145-dependent ubiquitination of SCAP. eLife..

[15] Costet P, Luo Y, Wang N (2000). Sterol-dependent transactivation of the human ABC1 promoter by LXR/RXR. J. Biol. Chem..

[16] Edwards PA, Kennedy MA, Mak PA (2002). LXRs; oxysterol-activated nuclear receptors that regulate genes controlling lipid homeostasis. Vascul. Pharmacol..

[17] Zelcer N, Hong C, Boyadjian R, Tontonoz P (2009). LXR regulates cholesterol uptake through Idol-dependent ubiquitination of the LDL receptor. Science.

[18] Jiang LY, Jiang W, Tian N (2018). Ring finger protein 145 (RNF145) is a ubiquitin ligase for sterol-induced degradation of HMG-CoA reductase. J. Biol. Chem..

[19] Schwartz AL, Ciechanover A (2009). Targeting proteins for destruction by the ubiquitin system: implications for human pathobiology. Annu. Rev. Pharmacol. Toxicol..

[20] Sharpe LJ, Cook ECL, Zelcer N, Brown AJ (2014). The UPS and downs of cholesterol homeostasis. Trends Biochem. Sci..

[21] Song B-L, Sever N, DeBose-Boyd RA (2005). Gp78, a membrane-anchored ubiquitin ligase, associates with Insig-1 and couples sterol-regulated ubiquitination to degradation of HMG CoA reductase. Mol. Cell..

[22] Jo Y, Lee PCW, Sguigna PV, DeBose-Boyd RA (2011). Sterol-induced degradation of HMG CoA reductase depends on interplay of two Insigs and two ubiquitin ligases, gp78 and Trc8. Proc. Natl. Acad. Sci. USA.

[23] Zelcer N, Sharpe LJ, Loregger A (2014). The E3 ubiquitin ligase MARCH6 degrades squalene monooxygenase and affects 3-hydroxy-3-methyl-glutaryl coenzyme A reductase and the cholesterol synthesis pathway. Mol. Cell Biol..

[24] Tsai YC, Leichner GS, Pearce MM (2012). Differential regulation of HMG-CoA reductase and Insig-1 by enzymes of the ubiquitin-proteasome system. Mol. Biol. Cell..

[25] Cha E, Fu Z-Y, Ma Y-T (2014). A novel polymorphism of the GP78 gene is associated with coronary artery disease in Han population in China. Lipids Health Disease.

[26] Osborne TF (1992). Single nucleotide resolution of sterol regulatory region in promoter for 3-hydroxy-3methylglutaryl coenzyme A reductase. J. Biol. Chem..

[27] Gidding SS, Whelton PK, Carey RM (2019). Writing a trustworthy hypertension guideline. J. Am. Coll. Cardiol..

[28] Shen JI, Nicholas SB, Williams S (2019). Evidence for and against ACC/AHA 2017 Guideline for target systolic blood pressure of < 130 mmHg in persons with Type 2 Diabetes. Curr. Cardiol. Rep..

[29] Pan S (2013). Appropriate body mass index and waist circumference cutoffs for categorization of overweight and central adiposity among Uighur adults in Xinjiang. PLoS ONE.

[30] Zhang M, Deng Q, Wang L (2018). Prevalence of dyslipidemia and achievement of low-density lipoprotein cholesterol target in Chinese adult: A nationally representative survey of 163,641 adults. Int. J. Cardiol..

[31] Luo J-Y, Ma Y-T, Yu Z-X (2014). Prevalence, awareness, treatment and control of dyslipidemia among adults in northwestern China: The cardiovascular risk survey. Lipids Health Dis..

[32] Gieger C, Radhakrishnan A, Cvejic A (2012). New gene functions in megakaryopoiesis and platelet formation. Nature.

[33] Bowes, J., Budu-Aggrey, A., Huffmeier, U. et al. Dense genotyping of immune-related susceptibility loci reveals new insights into the genetics of psoriatic arthritis. *Nat. Commun*. **6**, 6046 (2015). 10.1038/ncomms7046 PMC4327416 25651891

[34] Richardson, T.G., Sanderson, E., Palmer, T.M., *et al*. Evaluating the relationship between circulating lipoprotein lipids and apolipoproteins with risk of coronary heart disease: A multivariable Mendelian randomisation analysis. *PLoS Med*. **17**(3), e1003062 (2020). 10.1371/journal.pmed.1003062 PMC7089422 32203549

[35] Westra, H.J., Peters, M.J., Esko, T., *et al*. Systematic identification of trans eQTLs as putative drivers of known disease associations. *Nat. Genet*. **45**(10), 1238–1243 (2013). 10.1038/ng.2756 PMC3991562 24013639

